# Dermoscopic‐Pathologic Correlates of Invasiveness and Nevus Visibility in Nevus‐Associated Melanoma

**DOI:** 10.1111/exd.70333

**Published:** 2026-07-29

**Authors:** Marco Spadafora, Erica Baschieri, Simonetta Piana, Shaniko Kaleci, Giacomo Santandrea, Giovanni Pellacani, Caterina Longo

**Affiliations:** ^1^ Department of Surgery, Medicine, Dental Medicine and Morphological Sciences University of Modena and Reggio Emilia Modena Italy; ^2^ Azienda Unità Sanitaria Locale – IRCCS di Reggio Emilia Skin Cancer Center Reggio Emilia Italy; ^3^ Department of Dermatology Azienda Ospedaliero‐Universitaria Policlinico di Modena Modena Italy; ^4^ Pathology Unit Azienda Unità Sanitaria Locale ‐ IRCCS di Reggio Emilia Reggio Emilia Italy; ^5^ Dermatology Clinic, Department of Clinical Internal, Anesthesiological and Cardiovascular Sciences Sapienza University of Rome Rome Italy

**Keywords:** dermoscopy, diagnosis, melanoma, nevus‐associated melanoma, pathology

## Abstract

Nevus‐associated melanoma (NAM) is histologically defined by coexisting nevus and melanoma components, yet the nevus component is frequently not visible on dermoscopy.

We performed a retrospective, single‐centre observational study including histologically diagnosed NAMs (2011–2024). Dermoscopic images were assessed independently by two readers. Histopathology was systematically revised to quantify nevus proportion, size, and depth. Univariate and multivariable logistic regression evaluated associations with (a) invasive vs. in situ NAM and (b) dermoscopically visible vs. non‐visible nevus component.

Among 340 NAMs, 36.8% were in situ and 63.2% invasive. A dermoscopic nevus was visible in 45.6%. In multivariable analysis, nevus visibility under dermoscopy was independently associated with histopathologic features such as larger nevus size, particularly 2.1–4 mm (OR 2.5, 95% CI 1.3–4.6), 4.1–10 mm (OR 3.7, 95% CI 2.0–7.0), and > 10 mm (OR 5.3, 95% CI 1.4–20.0), whereas deep nevus location (OR 0.3, 95% CI 0.2–0.5) and ulceration (OR 0.2, 95% CI 0.1–0.6) reduced visibility. Invasive NAM was independently associated with a visible nevus component under dermoscopy (OR 2.5, 95% CI 1.7–3.7), shiny white structures (OR 5.0, 95% CI 2.6–9.4), negative pigment network (OR 1.9, 95% CI 1.2–3.2), and blue‐white veil (OR 8.3, 95% CI 4.5–15.4), whereas atypical pigment network and melanoma pigmentation were inversely associated with invasion.

Dermoscopic nevus visibility in NAM is mainly determined by nevus size and depth, while melanoma‐related changes, particularly ulceration, can obscure the nevus component. Dermoscopic markers of invasiveness should prompt timely management even when an associated nevus is suspected.

## Graphical abstract

Across 340 nevus‐associated melanomas, dermoscopic nevus visibility was independently determined by nevus depth, nevus size, and melanoma ulceration. Invasive NAM was associated with a visible nevus component, shiny white structures, and negative pigment network, whereas melanoma pigmentation, blue‐white veil, and atypical pigment network were inversely associated.

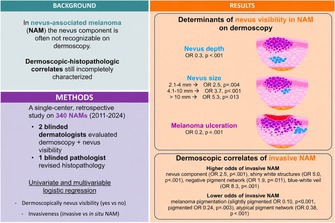

## 1 Introduction

Nevus‐associated melanoma (NAM) is defined histopathologically by the coexistence of nevus and melanoma components and still represents a controversial entity [1, 2, 3].

Even though a meta‐analysis has recently contributed to defining the prevalence of NAM, there is high heterogeneity in the literature on this value, depending on the study sample characteristics [4].

NAM more frequently appears on the trunk of younger patients with a higher nevus count than those developing de novo melanoma. Furthermore, patients with NAM have a fair skin phototype, non‐brown eyes, and a history of frequent sunburns during childhood [5]. Because of these phenotypic features, NAMs have been classified among non‐chronically sun‐damaged melanomas [6].

Some dermoscopic features that may facilitate the recognition of NAM have been reported, yet a reproducible and specific dermoscopic pattern has not been consistently identified to date [7, 8, 9, 10]. In particular, prior studies have described dermoscopic changes during follow‐up, as well as recurrent clues suggesting an underlying nevus component; however, the proposed signs remain heterogeneous across cohorts, limiting their diagnostic value [7, 8, 9, 10]. Of note, the nevus component in NAM could not always be identified by dermoscopy [2, 3, 9]. Although some studies suggest that invasive melanoma may overgrow and replace pre‐existing nevus cells, leading to misclassification as de novo melanoma when remnants are not visible [11], conversely, histopathologic nevus remnants may be present even when no nevus component is clinically or dermoscopically visible, and no clear evidence explains this discrepancy.

A few studies have investigated the correlation between specific histopathological features and the dermoscopic appearance of NAM [9, 10, 12, 13]. In particular, differences were observed between melanomas arising in association with a common acquired nevus versus a congenital nevus: the former typically develop at the periphery of a pre‐existing hypopigmented dermal nevus, whereas the latter more often arise within the centre of a congenital nevus. However, dermoscopic features have not yet been correlated with their histopathological counterparts. Furthermore, specific correlations between the reciprocal position of the melanoma and nevus components on histopathology and whether this influences the dermoscopic visibility of nevus components have not been reported.

In this study, we aimed to correlate dermoscopic findings with histopathologic architecture in NAM, focusing on the histopathologic determinants of dermoscopic nevus visibility and the dermoscopic features associated with histopathologic invasiveness.

## 2 Materials and Methods

A retrospective, single‐centre, observational study reviewed all melanomas histologically diagnosed as nevus‐associated. All patients were treated at the Skin Cancer Centre, and all histopathologic samples were analysed at the Pathology Unit of the Arcispedale Santa Maria Nuova in Reggio Emilia, Italy, between January 2011 and December 2024. Cases with no high‐quality dermoscopic images were excluded. Demographic data (age at diagnosis, sex, and site of the lesion) were retrieved from the Hospital Clinical Database.

Standardized polarized dermoscopic images were obtained with DermLite Photo (3Gen, San Juan Capistrano, CA, USA) mounted on a Canon G16 camera. Clinical and dermoscopic images were united for the assessment. All images were evaluated by two dermatologists with at least 5 years of expertise in dermoscopy (MS and EB). Images were analysed for the presence of selected clinical and dermoscopic criteria, Table S1; [8, 14]. Image sets were blinded to histopathological features other than NAM histotype. Evaluators were asked to indicate whether the nevus component was dermoscopically visible within the melanocytic lesion under dermoscopy and to describe dermoscopic features. Specifically, the nevus component was considered dermoscopically visible when the evaluators could identify a distinct area within the melanocytic lesion suggestive of a residual nevus component, based on the overall dermoscopic appearance compared with the melanoma component and/or based on specific dermoscopic clues of a benign melanocytic lesion. Cases with disagreement regarding the dermoscopic visibility of the nevus component were assessed by a third evaluator (CL). All histopathological samples were reviewed by an expert dermatopathologist (SP), who was blinded to dermoscopic nevus visibility, to identify specific criteria, as reported in Table S1; [15, 16].

### 2.1 Statistical Analysis

Statistical analyses were conducted using Stata software, version 18.0 (StataCorp LLC, College Station, TX, USA). All tests were two‐tailed, and a *p*‐value < 0.05 was considered statistically significant.

Dermoscopic and histologic variables were described using absolute frequencies and proportions. Descriptive analyses were reported separately for each evaluator (1–3) and for the histologic dataset.

Interobserver agreement was assessed using Cohen's kappa (*κ*) coefficient for all variables evaluated by more than one observer. The strength of agreement was interpreted according to Landis and Koch's criteria: < 0.00 = poor; 0.00–0.20 = slight; 0.21–0.40 = fair; 0.41–0.60 = moderate; 0.61–0.80 = substantial; > 0.80 = excellent.

Associations between variables and study outcomes were first explored using univariate logistic regression models. Specifically, analyses were performed to identify factors associated with (i) invasive versus in situ nevus‐associated melanoma, and (ii) dermoscopic visibility of the nevus component. Odds ratios (ORs) with 95% confidence intervals (95% CIs) were estimated. Given the number of dermoscopic and histopathologic variables examined, univariate analyses were considered exploratory and hypothesis‐generating.

To account for potential confounding and interdependence among variables, multivariable logistic regression models were subsequently constructed. Variables with clinical relevance or statistical significance in univariate analyses were considered for inclusion in the multivariable models. Model selection was guided by clinical plausibility and avoidance of collinearity.

Adjusted ORs with 95% CIs were reported.

## 3 Results

A total of 340 NAMs (340 patients) were included in the study. The mean age of the patients was 55.6 ± 14.4 years (range, 15–93). Most patients were male (60.6%). The trunk was the most frequent tumour location (67.4%), followed by the upper limbs (14.4%), lower limbs (9.7%), and head and neck (8.5%). Phototype II was predominant (56.5%). Among 340 melanomas, 125 (36.8%) were in situ, 136 (40.0%) were < 0.8 mm, 55 (16.2%) were 0.8–2.0 mm, and 24 (7.1%) were > 2.0 mm (Table S2). The nevus component was dermoscopically visible in 45.6% of cases. Fifty‐one melanomas were associated with a congenital nevus. Interobserver agreement was substantial to excellent across all assessed variables, with Cohen's *κ* values ranging from 0.78 to 1.00. Agreement was substantial for the evaluation of the nevus component (*κ* = 0.78) and near‐perfect for most dermoscopic features (up to *κ* = 1.00).

### 3.1 Univariable Logistic Regression Analysis: Invasive vs. In Situ NAM

Univariable analysis identified dermoscopic features associated with invasive NAM compared with in situ NAM (Table S3). The presence of a dermoscopically visible nevus component was significantly associated with invasive disease (OR 1.8, 95% CI 1.3–2.5; *p* < 0.001). Melanoma pigmentation exhibited an inverse association, with slightly pigmented (OR 0.06, 95% CI 0.03–0.1; *p* < 0.001) or pigmented melanomas (OR 0.2, 95% CI 0.08–0.4; *p* < 0.001) more frequently associated with in situ NAM.

**FIGURE 1:**
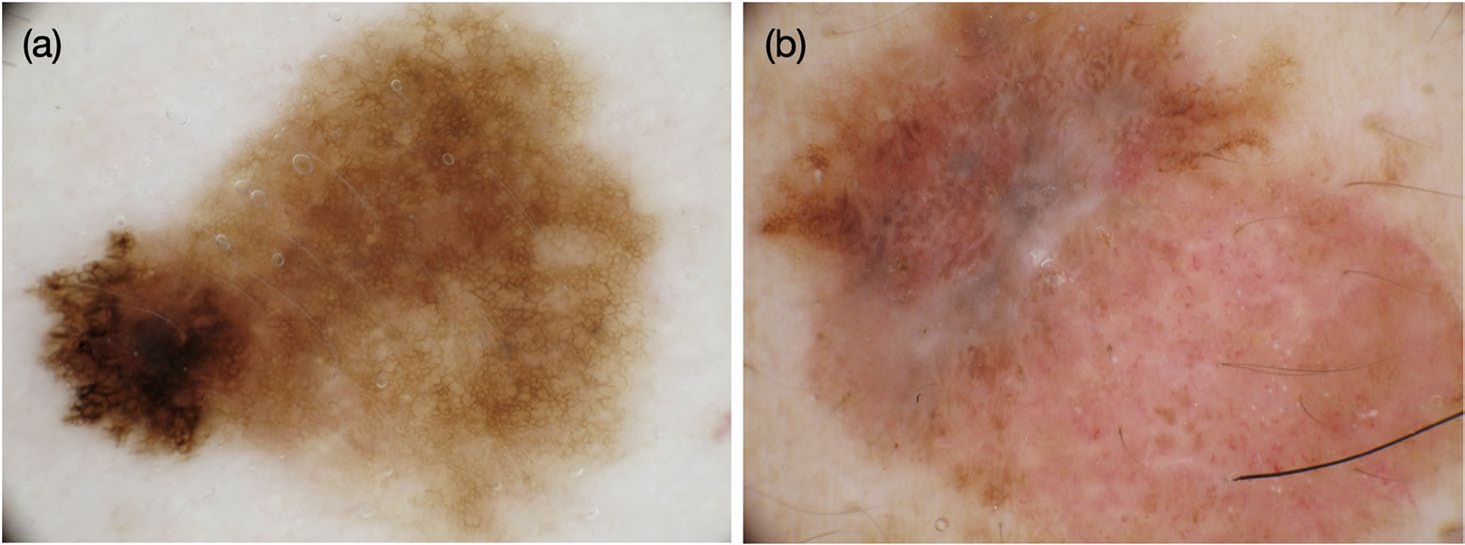
Dermoscopic images of two NAMs with a visible nevus component. (a) Dermoscopy of an in situ NAM on the arm of a 74‐year‐old female; on the right side, a slightly pigmented nevus with a reticular pattern; the melanoma component is located at the periphery on the left side, showing an atypical network. (b) Dermoscopy of an invasive NAM (Breslow thickness 1.0 mm) on the trunk of a 48‐year‐old male; on the right side, a hypopigmented compound nevus with a globular/cobblestone pattern, while on the left upper side we describe the melanoma component with a negative pigment network, regression structures, and shiny white structures.

Considering the predominant dermoscopic patterns, the following categories were associated with invasive melanomas (reticular pattern as the reference): globular/cobblestone (OR 10.4, 95% CI 4.7–22.7, *p* < 0.001), homogeneous (OR 3.0, 95% CI 1.9–4.8, *p* < 0.001), complex (OR 2.8, 95% CI 1.8–4.5, *p* < 0.001), multicomponent (OR 20.0, 95% CI 7.0–57.0, *p* < 0.001), and non‐specific (OR 15.8, 95% CI 6.6–37.9, *p* < 0.001) (Figure 1).

Several dermoscopic features also showed a significant association. The presence of an atypical pigment network was inversely correlated with invasive melanomas (OR 0.2, 95% CI 0.1–0.4, *p* < 0.001), while blue‐white veil (OR 10.3, 95% CI 5.9–18.0, *p* < 0.001), atypical vessels (OR 6.7, 95% CI 3.8–11.7, *p* < 0.001), irregular blotches (OR 2.6, 95% CI 1.9–3.6, *p* < 0.001), irregular dots and globules (OR 1.6, 95% CI 1.1–2.2, *p* < 0.005), irregular streaks (OR 1.9, 95% CI 1.2–3.1, *p* < 0.005), regression structures (OR 2.7, 95% CI 1.9–3.9, *p* < 0.001), negative pigment network (OR 1.9, 95% CI 1.3–2.9, *p* < 0.001), and shiny white structures (OR 11.2, 95% CI 6.3–19.9, *p* < 0.001) were all positively associated with invasive melanomas.

### 3.2 Multivariable Logistic Regression Analysis: Invasive vs. In Situ NAM

In the multivariable model (Table 1), the presence of a dermoscopically visible nevus component remained independently associated with invasive NAM (OR 2.5, 95% CI 1.7–3.7, *p* < 0.001). Melanoma pigmentation retained an inverse association with invasive disease, with both slightly pigmented (OR 0.10, 95% CI 0.04–0.25, *p* < 0.001) and pigmented melanomas (OR 0.24, 95% CI 0.09–0.61, *p* = 0.003) being less likely to be invasive compared with amelanotic/hypomelanotic lesions. Among dermoscopic structures, shiny white structures (OR 5.0, 95% CI 2.6–9.4, *p* < 0.001), negative pigment network (OR 1.9, 95% CI 1.2–3.2, *p* = 0.011), and blue‐white veil (OR 8.3, 95% CI 4.5–15.4, *p* < 0.001) were independently associated with invasive NAM, whereas the atypical pigment network remained inversely associated with invasiveness (OR 0.38, 95% CI 0.23–0.65, *p* < 0.001).

**TABLE 1:** Multivariable analysis of factors associated with invasive versus in situ nevus‐associated melanoma.

Variable	OR (95% CI)	*p*
Nevus component		
Absent	ref.	
Present	2.5 (1.7‐3.7)	**< 0.001**
Melanoma pigmentation		
Amelanotic/hypomelanotic	ref.	
Slightly pigmented	0.10 (0.04‐0.25)	**< 0.001**
Pigmented	0.24 (0.09‐0.61)	**0.003**
Shiny white structures		
Absent	ref.	
Present	5.0 (2.6‐9.4)	**< 0.001**
Negative pigment network		
Absent	ref.	
Present	1.9 (1.2‐3.2)	**0.011**
Atypical pigment network		
Absent	ref.	
Present	0.38 (0.23‐0.65)	**< 0.001**
Blue‐white veil		
Absent	ref.	
Present	8.3 (4.5‐15.4)	**< 0.001**

*Note:* The modelled outcome was invasive NAM, with in situ NAM as the reference outcome category. Bold values are statistically significant *p*‐value < 0.05.

### 3.3 Univariate Logistic Regression Analysis: Dermoscopically Visible Versus Non‐Visible Nevus Component

Univariable logistic regression identified histopathologic factors significantly associated with a dermoscopically visible nevus component on dermoscopy (Table S4).

**FIGURE 2:**
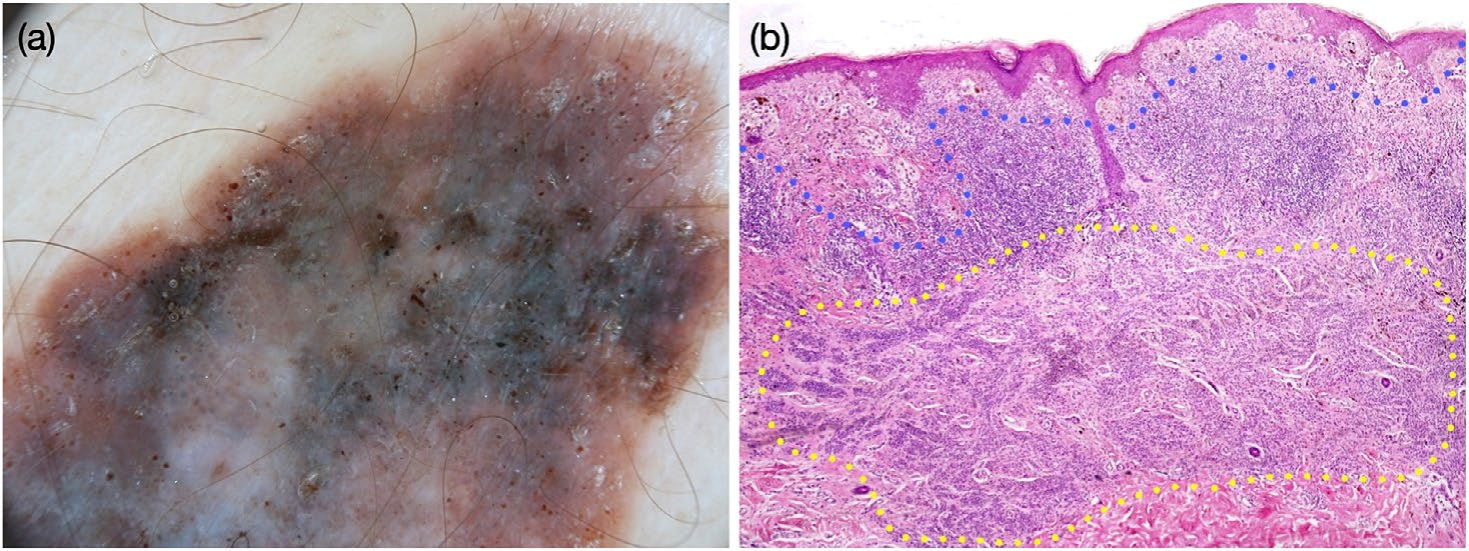
Invasive NAM (Breslow thickness 1.4 mm) on the back of a 44‐year‐old male. (a) Dermoscopic image showing a multicomponent pattern with a diffuse blue‐white veil and regression structures; the nevus component cannot be identified. (b) Histopathologic image (haematoxylin‐eosin staining, magnification × 40) showing dermal melanocytic nests corresponding to the nevus component (dotted yellow line) and a more superficial proliferation of atypical melanocytes corresponding to the melanoma component (dotted blue line). The relative depth of the nevus component and the presence of a blue‐white veil on dermoscopy could explain the absence of a dermoscopically visible nevus component.

**FIGURE 3:**
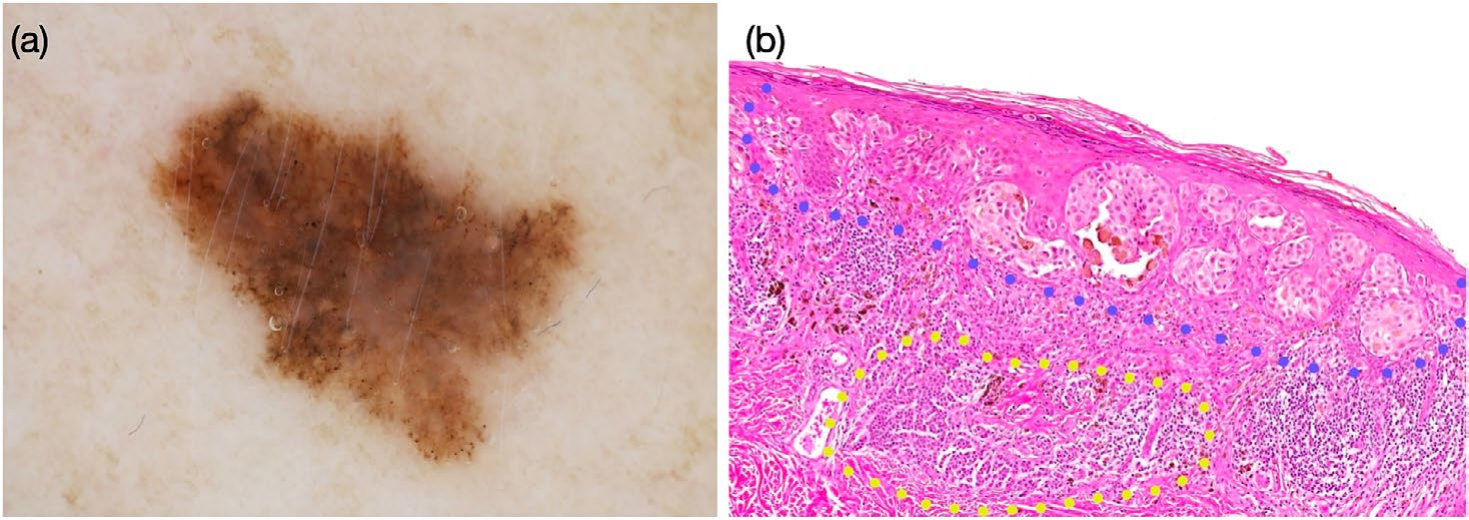
Invasive NAM (Breslow thickness 0.4 mm) on the back of a 39‐year‐old female. (a) Dermoscopic image showing a multicomponent pattern; the nevus component cannot be identified (b) histopathologic image (haematoxylin‐eosin staining, magnification × 100) showing a small aggregate of melanocytes corresponding to the nevus component (dotted yellow line) and a more superficial proliferation of atypical melanocytes corresponding to the melanoma component (dotted blue line). The small size of the nevus component could explain the absence of a dermoscopically visible nevus component.

Increasing relative nevus proportion within the overall melanocytic proliferation (nevus‐to‐melanoma size ratio) was strongly associated with a dermoscopically visible nevus component, with markedly higher odds of visibility for lesions with a 20%–50% and > 50% nevus component (OR 5.0 and 6.8, respectively; *p* < 0.001). Similarly, increasing nevus size correlated with a progressively higher probability of a dermoscopically visible nevus component (OR 2.0, for 1.1–2 mm, *p* = 0.002; OR 5.1, for 2.1–4 mm, *p* < 0.001; OR 7.1, 95% CI for 4.1–10 mm, *p* < 0.001; OR 11.7, 95% for >10 mm, *p* < 0.001). Nevus histotype, as well as the relative distribution of the nevus component on the horizontal plane (central vs. peripheral location in histopathology), did not show significant associations. Conversely, the relative depth of the nevus component in histopathology was inversely correlated with visibility (OR 0.3, 95% CI 0.2–0.4, *p* < 0.001) (Figures 2 and 3).

Nevus histotype was also evaluated in the univariate analysis, although this feature did not significantly influence the visibility of the nevus component under dermoscopy in our series (Table S4).

Among melanoma features, SSM lesions with a Breslow thickness < 0.8 mm (OR 2.0, 95% CI 1.2–3.3, *p* = 0.006) or 0.8–2 mm (OR 2.3, 95% CI 1.2–4.4, *p* = 0.012) were more likely to show a nevus component compared with in situ melanomas.

The superficial spreading subtype was also more frequently associated with a visible nevus (OR 1.8, 95% CI 1.3–2.5, *p* < 0.001), whereas nodular melanomas showed a non‐significant trend. Histopathological ulceration was inversely correlated with the visibility of the nevus component under dermoscopy (OR 0.3, 95% CI 0.1–0.9, *p* = 0.039).

In an exploratory univariable analysis, blue‐white veil was associated with lower odds of a dermoscopically visible nevus component (OR 0.2, 95% CI 0.1–0.5, *p* < 0.001, analysis not shown); however, this variable was not retained in the final multivariable model.

### 3.4 Multivariable Logistic Regression Analysis: Dermoscopically Visible Versus Non‐Visible Nevus Component

In multivariable logistic regression analysis, nevus size, relative depth of the nevus component, and ulceration remained independently associated with dermoscopic visibility of the nevus component (Table 2). Compared with nevi ≤ 1 mm, larger nevus remnants were associated with increased odds of dermoscopic visibility, particularly for lesions measuring 2.1–4 mm (OR 2.5, 95% CI 1.3–4.6; *p* = 0.004), 4.1–10 mm (OR 3.7, 95% CI 2.0–7.0; *p* < 0.001), and > 10 mm (OR 5.3, 95% CI 1.4–20.0; *p* = 0.013). A deep nevus component was independently associated with lower odds of a dermoscopically visible nevus component compared with a superficial component (OR 0.3, 95% CI 0.2–0.5; *p* < 0.001). Similarly, ulceration remained inversely associated with nevus component visibility (OR 0.2, 95% CI 0.1–0.6; *p* = 0.001).

**TABLE 2:** Multivariable analysis of factors associated with a dermoscopically visible nevus component in nevus‐associated melanoma.

Variable	OR (95% CI)	*p*
Nevus size (mm)		
≤ 1	ref.	
1.1–2	1.1 (0.6–2.0)	0.755
2.1–4	2.5 (1.3–4.6)	**0.004**
4.1–10	3.7 (2.0–7.0)	**< 0.001**
> 10 mm	5.3 (1.4–20.0)	**0.013**
Relative depth of nevus component		
Superficial	ref.	
Deep	0.3 (0.2–0.5)	**< 0.001**
Ulceration		
No	ref.	
Yes	0.2 (0.1–0.6)	**0.001**

*Note:* The modelled outcome was a dermoscopically visible nevus component, with the non‐visible nevus component as the reference outcome category. Bold values are statistically significant *p*‐value < 0.05.

## 4 Discussion

This single‐centre retrospective study provides dermoscopic‐histopathologic correlates in histologically confirmed NAMs, integrating quantitative histopathologic measurements with dermoscopic assessment of nevus visibility. By combining dermoscopic evaluation with standardised histopathologic revision, we identify architectural determinants that explain why the nevus component is frequently clinically and/or dermoscopically occult despite histologic association. Moreover, we obtained dermoscopic predictors of histopathologic invasiveness in NAMs. Notably, the multivariable analyses identified factors independently associated with invasive disease and those independently influencing dermoscopic visibility of the nevus component.

In our cohort, NAM predominantly involved fair‐skinned middle‐aged individuals, with a prevalence of males, and occurred most frequently on the trunk, with a high proportion of in situ and thin invasive tumours, supporting a phenotype that aligns with low cumulative sun‐damaged melanoma [17, 18, 19]. Previous studies have similarly shown that NAM is more commonly superficial spreading, trunk‐located, and diagnosed in younger patients and more frequently in males compared with de novo melanoma, reinforcing the concept of distinct individual and exposure features across these melanomas' developmental pathways [2, 5, 11].

### 4.1 Determinants of Dermoscopic Visibility of the Nevus Component

In our series, a nevus component was identified by dermoscopy in 45.6% of NAMs, indicating that more than half of histologically nevus‐associated tumours may be classified as ‘nevus‐negative’ based on dermoscopy alone. Prior works reported higher visibility of approximately two‐thirds, highlighting that dermoscopic visibility of the nevus component is variable even in expert settings and depends on study definitions and adjudication rules for ‘uncertain’ cases [2, 9].

Our more conservative classification, coding uncertain cases as “nevus not visible”, likely contributed to the lower visibility rate and may better reflect real‐world diagnostic management during routine clinical practice.

A major strength of our study is the identification of quantitative histopathologic predictors that could explain dermoscopic visibility of the nevus component.

The relative nevus proportion within the overall melanocytic proliferation was strongly associated with dermoscopic nevus visibility in the univariate analysis, suggesting that larger residual nevus components within the overall lesion are more likely to be dermoscopically visible. However, in the final multivariable model, independent predictors of nevus visibility were nevus size, relative depth of the nevus component, and ulceration.

Specifically, greater nevus size in millimetres on histopathologic examination correlated with progressively increased odds of dermoscopic visibility, supporting the intuitive concept that larger remnants are more likely to generate a distinct benign‐appearing dermoscopic ‘zone’ within the lesion. Importantly, this association remained significant in the multivariable analysis, where nevus remnants measuring more than 2.1 mm retained a progressively increased odds of dermoscopic visibility compared with smaller nevi. This finding confirms that the absolute histopathologic size of the residual nevus is an independent determinant of dermoscopic visibility, beyond other melanoma‐ or nevus‐related features.

Conversely, a deep, rather than superficial, nevus component substantially reduced dermoscopic visibility of the nevus component, providing a further histologic explanation for dermoscopically non‐visible NAMs in which nevus remnants are positioned deeply. This inverse relationship was also confirmed in the multivariable model, in which deep nevus remnants remained independently associated with lower odds of dermoscopic visibility. Accordingly, the anatomical relationship between melanoma and nevus components, together with nevus depth, likely determines whether dermoscopy can capture the ‘two‐component’ morphology, particularly in lesions arising in association with acquired dermal nevi [2]. These results complement prior qualitative observations that NAM may arise adjacent to acquired nevi, often leaving subtle or hypopigmented remnants, whereas NAM developing within/overlying congenital nevi may present more evident patterns [9].

Our findings also help clarify why a subset of histologic NAMs may appear clinically indistinguishable from de novo melanomas, particularly when nevus remnants are small or deep, and are coherent with the broader literature emphasizing that the absence of a dermoscopically visible nevus component does not preclude histologic nevus association [3, 11].

At the histopathologic level, it has also been proposed that invasive melanoma may overgrow or engulf pre‐existing nevus cells, potentially contributing to misclassification as de novo melanoma when nevus remnants are no longer visible [11]. This concept is relevant because the absence of a visible nevus component does not necessarily exclude a nevus‐associated origin, but may instead reflect a stage in which the melanoma component has prevailed over the pre‐existing nevus. However, this hypothesis should be interpreted cautiously and does not imply that all melanomas arise from nevi. Indeed, multicentre evidence showing persistent clinicopathological differences in thin melanomas suggests an intrinsic biological difference between NAM and de novo melanoma [11, 20].

Beyond nevus‐related architecture, melanoma‐related factors also influenced nevus visibility. In our cohort, invasive lesions were more likely to display a dermoscopically visible nevus component compared with in situ NAM, and the superficial spreading subtype was similarly associated with increased visibility. A plausible explanation is that, once invasion develops, the lesion more frequently expresses dermoscopic hallmarks of melanoma, which may enhance intra‐lesional heterogeneity and facilitate a ‘two‐component’ recognition by contrast with residual benign‐appearing nevus structures [10]. Adjunctively, the multivariable analysis showed that ulceration was independently associated with reduced visibility of the nevus component, suggesting that advanced melanoma‐related architectural disruption may obscure the residual nevus component.

Conversely, an exploratory univariable observation suggested that blue‐white veil may potentially reduce the perceptibility of residual nevus structures; a similar ‘masking’ effect of blue‐white veil has been previously reported in other dermoscopic settings, where it was shown to reduce the perceptibility of internal patterns by obscuring contrast within the lesion [21]. However, this hypothesis was supported only by an exploratory univariable observation and requires confirmation in further studies.

Overall, our findings indicate that the absolute size and superficial location of the nevus remnant are the main independent histopathologic determinants of dermoscopic visibility, whereas melanoma‐related architectural disruption, as reflected by ulceration, may independently reduce nevus visibility.

### 4.2 Dermoscopic Correlates of Invasive NAM

From our series, in situ NAM more typically displayed an atypical pigment network, in line with previous reports [3]. Conversely, in univariate analysis, invasive NAM was associated with several classic melanoma dermoscopic features, including blue‐white veil, atypical vessels, negative pigment network, regression structures, shiny white structures, and multicomponent or non‐specific global patterns [3]. However, only blue‐white veil, negative pigment network, and shiny white structures remained independently associated with invasive NAM in the multivariable model. Notably, in the multivariable analysis, the presence of a dermoscopically visible nevus component remained independently associated with invasive NAM.

Melanoma pigmentation was inversely related to Breslow thickness: both slightly pigmented and pigmented lesions were more often associated with lower Breslow categories. This association was confirmed in the multivariable model, where slightly pigmented and pigmented melanomas remained less likely to be invasive compared with amelanotic/hypomelanotic lesions. This finding reinforces the observation that amelanotic or hypomelanotic melanomas are more frequently associated with increased Breslow thickness, while pigmented lesions tend to be thinner [22, 23].

In univariate analysis, multicomponent and non‐specific patterns showed the highest odds of invasive NAM among the global dermoscopic patterns. This association may reflect greater intralesional heterogeneity in invasive lesions; however, neither pattern was retained in the final multivariable model and should therefore not be interpreted as an independent correlate of invasiveness. Nevertheless, this observation is conceptually consistent with the “intralesional comparative analysis” concept proposed by Borsari [10]. The authors showed that eccentric hyperpigmentation and dermoscopic color variegation, both reflecting intralesional asymmetry, markedly increased the likelihood of melanoma.

Notably, in comparative dermoscopy studies, NAM has been linked to globules, streaks, and negative pigment network, whereas de novo melanoma has been associated with blue‐white veil, suggesting partly divergent morphologic signatures [8, 12]. In our dataset, negative pigment network was associated with invasive NAM in both the univariate and multivariable models, indicating that this clue may also be associated with more advanced tumour invasiveness within nevus‐associated lesions [12, 21, 24]. Moreover, shiny white structures showed one of the strongest independent associations with invasive NAM, supporting their interpretation as dermoscopic markers of stromal change related to invasive growth [25]. Atypical pigment network remained inversely associated with invasiveness, consistent with its predominance in lesions where the junctional component is still the main driver of dermoscopic morphology [26].

In our series, regression structures were associated with invasive NAM, indicating that regression is not exclusive to early disease and may reflect dynamic tumour‐host interactions across stages.

Nevertheless, data focused on in situ melanoma suggest that dermoscopy alone remains unreliable for determining nevus association, but regression adjacent to an atypical lesion may raise suspicion for in situ NAM even when a nevus component is not dermoscopically visible [27].

Understanding dermoscopic correlates of histologic architecture may therefore improve early recognition and allow noninvasive stratification of risk during digital follow‐up.

### 4.3 Limitations

This study has several limitations. First, its retrospective, single‐centre design may limit external validity and may have introduced selection bias. Second, we excluded cases lacking high‐quality dermoscopic images, which may have affected the estimated rate of dermoscopic nevus visibility. Third, clinical variables (including palpability) were assessed from clinical/dermoscopic image sets rather than standardized in‐person examination, which may have led to measurement error and interobserver variability despite adjudication of discordant cases. Finally, the nevus areas or components identified on dermoscopy were not confirmed by a direct histopathologic mapping by corresponding tissue area.

In summary, our study bridges dermoscopic morphology with histopathologic architecture in NAM, demonstrating that nevus proportion, size, and depth are key determinants of dermoscopic nevus visibility. In multivariable analysis, nevus size and superficial localization remained independent determinants of visibility, whereas ulceration independently reduced the probability to visualising the associated nevus component. In parallel, melanoma‐related architectural changes may also influence nevus visibility, with ulceration independently associated with lower odds of a dermoscopically visible nevus component. Clinically, dermoscopic markers linked to invasiveness should prompt timely management even when an underlying nevus is suspected. Overall, these findings refine the diagnostic framework for NAM and underscore the value of integrating dermoscopic interpretation with histopathologic architecture to improve diagnostic accuracy and support appropriate, timely treatment decisions.

## Author Contributions

Conceptualization: Caterina Longo; data curation: Marco Spadafora, Erica Baschieri; formal analysis: Marco Spadafora, Shaniko Kaleci, Erica Baschieri, Simonetta Piana, Giacomo Santandrea; supervision: Caterina Longo; investigation: Marco Spadafora; writing – original draft: Marco Spadafora; writing – review and editing: Caterina Longo, Giovanni Pellacani, Simonetta Piana.

## Funding

Dr. Marco Spadafora was partially supported by the Italian Ministry of University and Research (MUR), through the PRIN 2022 Project “Cutaneous squamous cell carcinoma: stratifying high risk tumours with novel technologies” project code 2022MZEEZB (CUPE53D23013260006) funded by the National Recovery and Resilience Plan (PNRR), Italy, Mission 04 Component 2 Investment 1.1—NextGenerationEU.

## Ethics Statement

All procedures performed in this study were in accordance with the ethical standards of the institutional and national research committee and with the 1964 Helsinki Declaration and its later amendments or comparable ethical standards. The patients in this manuscript have given written informed consent to publication of their case details. This study was approved by the Institutional Review Board of Azienda Unità Sanitaria Locale—IRCCS di Reggio Emilia, Italy (CE: 1345/2020/OSS/IRCCSRE).

## Conflicts of Interest

The authors declare no conflicts of interest.

## Supporting information


**Table S1:** Clinical, dermoscopic, and histopathological criteria.
**Table S2:** Demographic and clinical features of the enrolled population.
**Table S3:** Univariable analysis of factors associated with invasive versus in situ nevus‐associated melanoma.
**Table S4:** Univariable analysis of factors associated with a dermoscopically visible nevus component in nevus‐associated melanoma.

## Data Availability

The data that support the findings of this study are available from the corresponding author upon reasonable request.
