# Supplementary material for: Dermoscopic‐Pathologic Correlates of Invasiveness and Nevus Visibility in Nevus‐Associated Melanoma
Source: Exp Dermatol. 2026 Jul 29;35(8):e70333. doi: 10.1111/exd.70333 (PMC13420791; doi:10.1111/exd.70333)
Supplement: Supplementary file 1 — Table S1: Clinical, dermoscopic, and histopathological criteria. Table S2: Demographic and clinical features of the enrolled population. Table S3: Univariable analysis of factors associated with invasive versus in situ nevus‐associated melanoma. Table S4: Univariable analysis of factors associated with a dermoscopically visible nevus component in nevus‐associated melanoma. [file EXD-35-e70333-s001.docx]

**SUPPLEMENTARY MATERIAL**

**Supplementary Table 1** – Clinical, dermoscopic, and histopathological criteria

| **Nevus characteristics** | **Definition** |
| --- | --- |
| ***Nevus component*** | The dermoscopic visibility of a residual nevus component, recorded as visible or non-visible |
| ***Nevus palpability*** | Assessed from clinical pictures as flat; papular-slightly palpable; nodular-plaque |
| ***Nevus pigmentation*** | Defined as amelanotic/hypomelanotic, slightly pigmented, pigmented |
| ***Nevus predominant dermoscopic pattern*** | Defined as reticular, globular/cobblestone, homogeneous, complex/multicomponent |
| **Melanoma characteristics** | **Definitions** |
| ***Melanoma detection*** | The distinction between the nevus and the melanoma by differences in color, palpability, shape, or a combination of these features |
| ***Melanoma localization*** | Melanoma localization in relation to the nevus, recorded as peripheral or central |
| ***Melanoma palpability*** | Assessed from clinical pictures as flat, papular-slightly palpable, nodular-plaque |
| ***Melanoma pigmentation*** | Defined as amelanotic/hypomelanotic, slightly pigmented, pigmented |
| ***Melanoma predominant dermoscopic pattern*** | Defined as reticular, globular/cobblestone, homogeneous, complex (referring to the combination of two of the above-mentioned patterns), multicomponent (all of the mentioned pattern) or nonspecific. |
| ***Atypical network*** | A pigment network showing marked variation in line thickness and color, with network holes of heterogeneous size and shape. |
| ***Blue-white veil*** | Irregular structureless area of confluent blue pigmentation overlaid by a whitish ‘ground glass’ film |
| ***Atypical vessels*** | Irregular, disorganized, or polymorphous vascular structures |
| ***Irregular dots/globules*** | Round to oval pigmented structures of variable size and color (brown to black), asymmetrically distributed throughout the lesion or at the periphery of the lesion |
| ***Irregular streaks*** | \| Peripheral linear (“finger-like” structures) of varying thickness and length, irregularly distributed at the lesion periphery \| \| --- \| |
| ***Irregular blotches*** | Asymmetrically distributed, dark pigmented areas (black, brown, or gray) without specific structure |
| ***Regression structures*** | Areas showing white scar-like depigmentation and/or blue-gray “pepper-like” granularity |
| ***Negative pigment network*** | A reversed network pattern where hypopigmented lines form the “cords” and darker areas fill the “holes.” |
| ***Shiny white streaks*** | Bright, orthogonal, or parallel white lines visible only under polarized dermoscopy |
| **Histopathological criteria** | **Definition** |
| ***Nevus histotype*** | Defined as junctional compound, intradermal, congenital intradermal) |
| ***Nevus/melanoma (%)*** | It represents the ratio of the nevus size to the melanoma size (<10%; 10-20%; 20-50%; >50%) |
| ***Nevus size (mm)*** | Nevus size, expressed in mm (<=1.0mm; 1.1-2.0mm; 2.1-4.0mm; 4.1-10mm; >10mm) |
| ***Relative distribution of nevus component*** | The localization of the nevus in relation to the melanoma on a horizontal plane (central; peripheral) |
| ***Relative depth of nevus component*** | The depth of the nevus assessed on a tangential plane in relation to the melanoma (superficial; deep) |
| ***Melanoma histotype*** | Defined as in situ, superficial spreading and nodular |
| ***Breslow thickness (mm)*** | Represents the vertical depth of melanoma invasion measured in millimeters, taken from the top of the granular layer of the epidermis (or from the base of any ulceration) to the deepest point of tumor invasion |
| ***Ulceration*** | The absence of an intact epidermis overlying the primary melanoma |

**Supplementary Table 2 –** Demographic and clinical features of the enrolled population

| **Variable** | **N=340** | **Proportion (%)** |
| --- | --- | --- |
| **Age, Mean ± SD(range)** | 55.6 ±14.4(15-93) | |
| **Sex** |  |  |
| Female | 134 | 39,4 |
| Male | 206 | 60,6 |
| **Location** |  |  |
| Head and Neck | 29 | 8,5 |
| Upper limb | 49 | 14,4 |
| Trunk | 229 | 67,4 |
| Lower limb | 33 | 9,7 |
| **Phototype** |  |  |
| I | 88 | 25,9 |
| II | 192 | 56,5 |
| III | 60 | 17,6 |
| **Melanoma Breslow thickness (mm)** |  |  |
| In situ | 125 | 36.8 |
| <0.8 | 136 | 40 |
| 0.8-2 | 55 | 16.2 |
| >2 | 24 | 7.1 |

**Supplementary Table 3 –** Univariable analysis of factors associated with invasive versus in situ nevus-associated melanoma

| **Variable** | **OR** (CI 95%) | **p-value** |
| --- | --- | --- |
| **Nevus component** |  |  |
| absent | ref. |  |
| present | 1.8 (1.3-2.5) | **<0.001** |
| **Nevus pigmentation** |  |  |
| amelanotic/hypomelanotic | ref. |  |
| slightly pigmented | 1.4 (0.8-2.3) | 0.171 |
| pigmented | 1.9 (0.4-9.5) | 0.441 |
| **Nevus predominant dermoscopic pattern** |  |  |
| reticular | ref. |  |
| globular/cobblestone | 1.5 (0.8-2.7) | 0.222 |
| homogeneous | 0.6 (0.3-1.1) | 0.124 |
| complex/multicomponent | 2.2 (0.8-6.3) | 0.134 |
| **Melanoma location** |  |  |
| peripheral/eccentric | ref. |  |
| central | 0.9 (0.4-1.8) | 0.747 |
| **Melanoma pigmentation** |  |  |
| amelanotic/hypomelanotic | ref. |  |
| slightly pigmented | 0.06 (0.03-0.1) | <0.001 |
| pigmented | 0.2 (0.08-0.4) | <0.001 |
| **Melanoma predominant dermoscopic pattern** |  |  |
| reticular | ref. |  |
| globular/cobblestone | 10.4 (4.7 -22.7) | <0.001 |
| homogeneous | 3.0 (1.9-4.8) | <0.001 |
| complex | 2.8 (1.8-4.5) | <0.001 |
| multicomponent | 20.0 (7.0-57.0) | <0.001 |
| nonspecific | 15.8 (6.6-37.9) | <0.001 |
| **Nevus histotype** |  |  |
| junctional | ref. |  |
| compound | 1.3 (0.2-8.3) | 0.758 |
| intradermal | 0.2 (0.04-1.2) | 0.077 |
| intradermal congenital | 0.4 (0.08-2.2) | 0.302 |
| **Atypical pigment network** |  |  |
| absent | ref. |  |
| present | 0.2 (0.1-0.4) | **<0.001** |
| **Blue-white veil** |  |  |
| absent | ref. |  |
| present | 10.3 (5.9-18.0) | **<0.001** |
| **Atypical vessels** |  |  |
| absent | ref. |  |
| present | 6.7 (3.8-11.7) | **<0.001** |
| **Irregular blotches** |  |  |
| absent | ref. |  |
| present | 2.6 (1.9-3.6) | **<0.001** |
| **Irregular dots/globules** |  |  |
| absent | ref. |  |
| present | 1.6 (1.1-2.2) | 0.005 |
| **Irregular streaks** |  |  |
| absent | ref. |  |
| present | 1.9 (1.2-3.1) | 0.005 |
| **Regression structures** |  |  |
| absent | ref. |  |
| present | 2.7 (1.9-3.9) | **<0.001** |
| **Negative pigment network** |  |  |
| absent | ref. |  |
| present | 1.9 (1.3-2.9) | **0.001** |
| **Shiny white structures** |  |  |
| absent | ref. |  |
| present | 11.2 (6.3-19.9) | **<0.001** |

The modeled outcome was invasive NAM, with in situ NAM as the reference outcome category.

**Supplementary Table 4 –**Univariable analysis of factors associated with a dermoscopically visible nevus component in nevus-associated melanoma

| **Variable** | **OR (95% CI)** | **p-value** |
| --- | --- | --- |
| **nevus/melanoma (%)** |  |  |
| <10 | ref. |  |
| 10–20 | 0.9 (0.6-1.5) | 0.880 |
| 20–50 | 5.0 (3.2-7.8) | <0.001 |
| >50 | 6.8 (4.2-11.1) | <0.001 |
| **Nevus size (mm)** |  |  |
| ≤1 | ref. |  |
| 1.1–2 | 2.0 (1.3 -3.2) | 0.002 |
| 2.1–4 | 5.1 (3.2-8.1) | <0.001 |
| 4.1–10 | 7.1 (4.4 -11.4) | <0.001 |
| >10 mm | 11.7 (3.7-37.1) | <0.001 |
| **Nevus histotype** |  |  |
| Junctional | ref. |  |
| Compound | 1.3 (0.2-8.3) | 0.758 |
| Intradermal | 0.2 (0.04-1.2) | 0.077 |
| Congenital intradermal | 0.4 (0.08-2.2) | 0.302 |
| **Relative distribution of nevus component** |  |  |
| Peripheral | ref. |  |
| Central | 0.8 (0.6-1.1) | 0.166 |
| **Relative depth of nevus component** |  |  |
| Superficial | ref. |  |
| Deep | 0.3 (0.2–0.4) | **<0.001** |
| **Breslow thickness (mm)** |  |  |
| In situ | ref. |  |
| <0.8 | 2.0 (1.2–3.3) | **0.006** |
| 0.8–2 | 2.3 (1.2–4.4) | **0.012** |
| >2 mm | 0.7 (0.3–1.9) | 0.521 |
| **Melanoma histotype** |  |  |
| In situ | ref. |  |
| SSM | 1.8 (1.3-2.5) | <0.001 |
| **Ulceration** |  |  |
| No | ref. |  |
| Yes | 0.3 (0.1–0.9) | **0.039** |
|  |  |  |

The modeled outcome was a dermoscopically visible nevus component, with the non-visible nevus component as the reference outcome category.
